# Elucidating a fresh perspective on the interplay between exosomes and rheumatoid arthritis

**DOI:** 10.3389/fcell.2023.1177303

**Published:** 2023-04-28

**Authors:** Jianan Zhao, Binbin Zhang, Wanting Meng, Jing Hu

**Affiliations:** ^1^ Department of Nephropathy, The Seventh People’s Hospital Affiliated to Shanghai University of Traditional Chinese Medicine, Shanghai, China; ^2^ Department of Rheumatology, Shanghai Guanghua Hospital, Shanghai University of Traditional Chinese Medicine, Shanghai, China; ^3^ Guanghua Clinical Medical College, Shanghai University of Traditional Chinese Medicine, Shanghai, China; ^4^ Arthritis Institute of Integrated Traditional and Western Medicine, Shanghai Chinese Medicine Research Institute, Shanghai, China; ^5^ Zhejiang University of Traditional Chinese Medicine, Hangzhou, China; ^6^ Department of Translational Medicine Platform, The Affiliated Hospital of Hangzhou Normal University, Hangzhou, China; ^7^ Academy of Integrative Medicine, Shanghai University of Traditional Chinese Medicine, Shanghai, China

**Keywords:** rheumatoid arthritis, exosomes, microbial communities, blood, mesenchymal stem cells, fibroblast-like synovial cells, gut microbe-derived exosomes

## Abstract

Rheumatoid arthritis (RA) is a chronic autoimmune disease characterized by chronic synovitis and the destruction of bones and joints. Exosomes are nanoscale lipid membrane vesicles originating from multivesicular bodies and are used as a vital means of intercellular communication. Both exosomes and the microbial community are essential in RA pathogenesis. Multiple types of exosomes from different origins have been demonstrated to have effects on various immune cells through distinct mechanisms in RA, which depend on the specific cargo carried by the exosomes. Tens of thousands of microorganisms exist in the human intestinal system. Microorganisms exert various physiological and pathological effects on the host directly or through their metabolites. Gut microbe-derived exosomes are being studied in the field of liver disease; however, information on their role in the context of RA is still limited. Gut microbe-derived exosomes may enhance autoimmunity by altering intestinal permeability and transporting cargo to the extraintestinal system. Therefore, we performed a comprehensive literature review on the latest progress on exosomes in RA and provided an outlook on the potential role of microbe-derived exosomes as emerging players in clinical and translational research on RA. This review aimed to provide a theoretical basis for developing new clinical targets for RA therapy.

## Introduction

Rheumatoid arthritis (RA) is a chronic autoimmune disease characterized by chronic persistent synovitis, systemic inflammation, and the presence of autoantibodies such as rheumatoid factor and anti-cyclic citrullinated peptide (anti-CCP) (Lee and Weinblatt, 2001; Scott et al., 2010). Untreated RA may lead to progressive disability and reduced quality of life, profoundly affecting the patient’s and family’s physical and mental health and increasing the burden on the whole family and society (Meier et al., 2013). For the clinical management of patients with RA, the first treatment principle is early detection, early intervention, and clinical remission with immunosuppression under the guidance of a physician (Meier et al., 2013). Currently, the main therapeutic drugs for RA include disease-modifying anti-rheumatic drugs, non-steroidal anti-inflammatory drugs, hormonal drugs, and new biologics to improve the treatment of the disease. Although certain clinical efficacy has been achieved, due to the complex factors of the disease itself, some patients still show no or poor/little response; therefore, the discovery of new disease targets and the development of clinical drugs with this is a top priority (Zhao et al., 2021).

RA risk factors include genetic, environmental, metabolic, immune, and microbial interactions, which lead to abnormal pathological features (Scott et al., 2010). Among these, multiple mechanisms of microbial community disorders are considered important pathological mechanisms (de Oliveira et al., 2017). Humans are multicellular organisms that co-exist as macroscopic beings with a diverse symbiotic microbial community, which is conservatively estimated to substantially exceed the number of constituent cells of humans (approximately 100 trillion). The microbial community also expresses more unique genes in its genome (Belkaid and Harrison, 2017). The existence of a large number of microbial communities contributes to the physiological functions of the body through a variety of mechanisms. The interaction between the microbial community and the host immune system is crucial to the local and systemic immune responses. Maintaining the balance of the microbiota is an essential factor for maintaining homeostasis (Lu et al., 2022). The primary association between gut microbes and RA is that preclinical and clinical patients with RA have abnormal changes in gut microbiota and gut microbial metabolites. These abnormal changes in microbial communities and metabolites may lead to abnormal immune recognition responses mediating the autoimmune response in RA.

The International Society for Extracellular Vesicles proposed Minimal Information for Studies of Extracellular Vesicles (“MISEV”) guidelines suggest the use of “EVs” to generally denote a heterogeneous extracellular vesicle population, and “exosomes” are defined as small extracellular vesicles (Cocucci and Meldolesi, 2015; Théry et al., 2018). Multiple sources of extracellular vesicles exist in RA, which exerts effects on various immune cells. As for the extracellular vesicles themselves, they are an emerging and widely researched topic; they are being used as a diagnostic and therapeutic tool in the treatment of various diseases as a critical mechanism of intercellular communication, organ homeostasis, and disease (Kalluri and LeBleu, 2020). Microbe-derived exosomes are emerging stars in the field of exosomes that may play a crucial role in liver diseases. Their effects may include anti-inflammatory, intestinal barrier restorative, and immune modulatory effects, among other mechanisms (Zhang et al., 2022). Given the link between microbes and exosomes and RA, we present a summary and review of both and further examine the role of microbe-derived exosomes in the clinical management of RA to provide new theoretical support and options for the clinical management of RA.

## Overview of the progress of exosomes and RA

Exosomes are defined as a set of vesicle-like structures released by all cell types, whose contain and exposure at their membrane surface protein and lipid components as well as nucleic acids originating from their original cell (Turpin et al., 2016). Exosomes are unable to replicate independently and are known by various names, such as microvesicles and microparticles (Miao et al., 2022). The extracellular vesicle can primarily be classified into two major categories, namely, ectosomes and exosomes. Ectosomes represent vesicles that result from the direct outward protrusion of the plasma membrane, generating entities such as microvesicles, microparticles, and large vesicles with sizes ranging from approximately 50 nm to 1 μm in diameter. Conversely, exosomes originate from endosomes and exhibit sizes within the range of approximately 40 nm–160 nm in diameter, with an average size of around 100 nm (Kalluri and LeBleu, 2020). The production of exosomes involves a double invagination of the plasma membrane and the creation of intracellular multivesicular bodies (MVBs) containing intraluminal vesicles (ILVs). The ILVs are eventually released as exosomes, which have a diameter ranging from approximately 40 nm–160 nm, through the process of MVB fusion with the plasma membrane and subsequent exocytosis (Kalluri and LeBleu, 2020). In accordance with the guidelines of The International Society for Extracellular Vesicles 2018 and consistency throughout the text, we use exosomes consistently throughout the text (Cocucci and Meldolesi, 2015; Théry et al., 2018). Exosomes mainly mediate intercellular messaging and are involved in numerous physiological processes, such as inflammation, immune response, angiogenesis, cellular stress, cellular senescence, cell proliferation, and differentiation (Turpin et al., 2016). Transcriptomic analysis revealed that exosomes transport a variety of cargoes, including many functional small coding miRNAs, messenger RNA (mRNA), and non-coding RNA (ncRNAs) (Lee et al., 2007; Kaparakis-Liaskos and Ferrero, 2015). Proteomic analysis has demonstrated that exosomes also transport other cargo, including membrane-bound proteins, enzymes (e.g., autolysins), toxins, polysaccharides, and peptidoglycans (Lee et al., 2007; Kaparakis-Liaskos and Ferrero, 2015). There exist different patterns of extracellular vesicle in RA. For instance, Hirotaka et al. found significant differences in the serum exosomes protein profile between active RA patients, osteoarthritis (OA) patients, and healthy individuals. Specifically, RA patients exhibited higher levels of TLR3 and pro-neuregulin-3 proteins, which may reflect the hyperactive state of RA fibroblast-like synoviocytes (FLS) (Tsuno et al., 2018). Proteomic analysis of plasma exosomes from RA patients and healthy controls identified 32 upregulated proteins and 5 downregulated proteins. Bioinformatics analysis indicated that these differentially expressed proteins interact with each other and mainly participate in inflammation pathways affecting RA (Qin et al., 2021). Results from a systematic review and meta-analysis showed that the total number of exosomes in both plasma and synovial fluid was increased in RA patients compared to healthy individuals, which is mainly associated with the release of cytokines and chemokines(Schioppo et al., 2021).Compared with osteoarthritis, gout, and axial spondyloarthritis, it was found that 28 proteins were uniquely upregulated in exosomes derived from RA synovial fluid. Bioinformatics analysis indicated their significant involvement in “serine-type endopeptidase inhibitor activity” and “complement and coagulation cascades” (Huang et al., 2022a). Therefore, We demonstrate the therapeutic potential of exosomes from different sources by elucidating their effects on different immune cell populations in RA.

## Potential effects of different sources of exosomes on fibroblast-like synovial cells in RA

RA fibroblast-like synovial cells (FLS) are a crucial population responsible for abnormal synovial proliferation, inflammation, and bone destruction. Exosomes originating from RA FLS themselves are shown to promote their abnormal proliferation and synovial hyperplasia by carrying certain substances secreted by the cells. RA FLS is an essential group of cells in RA that causes abnormal synovial proliferation, inflammation, and bone destruction. Exosomes from RA FLS promote their abnormal proliferation and synovial proliferation by carrying some substances secreted by themselves. For example, Exosomes from RA FLS contain membrane-bound forms of tumor necrosis factor-α (TNF-α), which promotes the activation of nuclear factor kappa-light-chain-enhancer of activated B cells (NF-KB) and induction of membrane-type matrix metalloproteinase (MMP)-1 in RA FLS (Zhang et al., 2006). RA FLS overexpress and releases cluster of differentiation (CD) 13, which acts mainly as an intracellular soluble molecule and is encapsulated in exosomes, enhancing RA FLS proliferation and migration by acting on itself. Inhibition of CD13 can inhibit this effect (Morgan et al., 2016). Transforming growth factor beta (TGF-β) stimulates FLS to produce an inhibitor of DNA binding 1, which is passed to endothelial progenitor cells and human dermal microvascular endothelial cells and activates the c-Jun N-terminal kinases (JNKs) signaling pathway to promote angiogenesis through exosomes from RA FLS (Edhayan et al., 2016). Some exosomes from other sources have different effects on promoting and inhibiting RA FLS proliferation and inflammation, which depend on the functional roles of the cargo types carried by the exosomes. For example, the peripheral blood mononuclear cell-derived exosome *lncRNA* nuclear-enriched abundant transcript 1 (*NEAT1*) targets and regulates the miR-23a/mouse double minute 2 homolog (MDM2)/sirtuin 6 (SIRT6) axis to promote proliferation and inflammation in RA FLS (Rao et al., 2020). However, T cells release large amounts of derived exosomes, *miR-204-5p*, to transfer to FLS and inhibit cell proliferation (Wu et al., 2022). It is noteworthy that exosomes derived from mesenchymal stem cells have been extensively studied and proven to be beneficial for RA, including: i) Reducing vascular endothelial growth factor (VEGF) expression and angiogenesis: For example, Synovial mesenchymal cell-derived exosomes inhibit *miR-485-3p*/signal transducer and activator of transcription 3 (STAT3) by transmitting *circRNA-circEDIL3* to reduce *VEGF* expression and angiogenic activity in RA FLS induced *in vitro* and reduce the extent of arthritis in collagen-induced arthritis (CIA) models (Zhang et al., 2021). Similarly, mesenchymal stem cell-derived *miR-150-5p* targeting membrane-type matrix metalloproteinase 14(*MMP14*), and *VEGF* reduced angiogenic activity and clinical arthritis scores in arthritic mice (Chen et al., 2018). ii) Inhibiting synovial inflammation, synovial hyperplasia, and bone destruction. For example, human umbilical cord mesenchymal stem cells (MSCs)-derived exosome *miR-140-3p* inhibited inflammation, oxidative stress, and fibrotic response and suppressed synovial proliferation by silencing serum- and glucocorticoid-induced kinase 1 (*SGK1*) in CIA models (Huang et al., 2022b). MSCs-derived exosomes of *circFBXW7* and *miR-320a* can inhibit cell proliferation, migration, inflammatory response, and bone damage in RA FLS by directly targeting *miR-216a-3p* to upregulate histone deacetylase 4 (*HDAC4*) and C-X-C motif chemokine 9 (*CXCL9*) (Meng and Qiu, 2020; Chang and Kan, 2021). Chondrogenic bone marrow mesenchymal stem cell-derived exosomes directly inhibit RA FLS pro-inflammatory factors, MMP production, and activation of mitogen-activated protein kinase (MAPK) and NF-KB pathways and ameliorate arthritis in CIA mice (Ma et al., 2022).

## Potential effects of different sources of exosomes on T helper 17 cells, regulatory T cells, and regulatory B cells in RA

T helper 17 (Th17) cells, a subset of T cells, are a pro-inflammatory cell population in RA. Exosomes from different sources can affect disease progression by influencing the proliferation of CD4^+^ T cells and their differentiation towards Th17 cells. For example, autoimmune T cells engulfing RA FLS-derived exosomes cause increased resistance to apoptosis and prolong the survival time (Zhang et al., 2006). The *lncRNA NEAT* in RA blood exosomes promotes CD4^+^ T cell proliferation and Th17 cell differentiation by regulating the *miR-144-3p*/*rho* associated coiled-coil containing protein kinase 2(ROCK2)/WNT axis for disease progression (Liu et al., 2021). Interleukin (IL)-17, a pro-inflammatory cytokine secreted by Th17 cells, is an important pro-inflammatory factor in RA. Exosomes derived from human gingival mesenchymal stem cells inhibited IL-17A secretion and promoted the secretion of the anti-inflammatory factor IL-10, reduced bone erosion, and improved arthritis in CIA models and *in vitro* CD4^+^ T cell co-culture models (Tian et al., 2022). This further supports the link between the mouth and joints. A reduction in T cell differentiation towards the regulatory T (Treg) cells is a key factor in inflammation in RA, as Treg cells are an important cell population that suppresses Th17 differentiation. The effects of exosomes from different sources on Treg cell may be bidirectional. On the one hand, they may promote inflammation by inhibiting Treg cells. For example, exosomes derived from RA FLS *lncRNA* HOXA distal transcript antisense RNA (*HOTTIP*) negatively regulate *miR-1908-5p* and positively regulate *STAT3*, leading to upregulation of forkhead box P3 (*FOXP3*) and retinoid-related orphan receptor gamma (*RORγt*) expression and Th17/Treg cell ratio imbalance (Yao et al., 2021). Similarly, the RA joint cavity exhibited an overall hypoxic microenvironment owing to the over-proliferation of FLS. *miR-424* in exosomes derived from RA FLS significantly induced Th17 differentiation and inhibited Treg cells differentiation under hypoxic conditions (Ding et al., 2020). On the other hand, they may promote the suppression of inflammation by enhancing Treg cells. For example, exosomes from MSCs can significantly suppress T- and B cell immune responses, increase Treg cells *in vitro*, and significantly suppress inflammatory responses in CIA models (Cosenza et al., 2018). *miR-146a/miR-155* in MSCs exosomes suppress aberrant immune responses by increasing *FOXP3*, *RORγt, TGF-β, IL-10, IL-17,* and *IL-6* expression and increasing the Treg cell population in CIA models (Tavasolian et al., 2020).

In addition, autoimmune B cells are the source of many autoantibodies, and regulatory B (Breg) cells suppress inflammation by secreting IL-10 and other anti-inflammatory factors in RA. Exosomal prostaglandin E2 secreted by granulocytic myeloid-derived suppressor cells can significantly upregulate glycogen synthase kinase-3 beta (*GSK-3 beta*), and *adenosine triphosphate response element-binding protein* phosphorylation can promote IL-10+ Breg production to suppress inflammation in CIA models (Wu et al., 2020).

## Potential effects of different sources of exosomes on osteoblasts, chondrocytes, and osteoclasts in RA

The destruction of osteoblasts and the excessive production of osteoclasts are important factors in bone destruction in RA. On the one hand, some exosomes may promote the process of bone destruction. Reacher found that the levels of exosomal receptor activator of nuclear factor kappa-B ligand (RANKL) isolated from the synovial fluid of patients with RA were significantly higher than those of patients with several other types of arthritis and induced higher numbers of osteoclasts involved in bone destruction (Song et al., 2021). Smoking is considered a risk factor for RA (Zhao et al., 2021). Cigarette smoke promotes exosomal miR-132 secretion by Th17 cells, downregulating cyclooxygenase-2 (COX2) to promote osteoclastogenesis, and knockdown of miR132 in an arthritis model reduced the number of osteoclasts (Donate et al., 2021). Exosomes originating from RA FLS affect the RANKL/RANK/osteoprotegerin (OPG) pathway by transporting *miR-106b* to chondrocytes and inhibiting pyruvate dehydrogenase kinase isozyme 4 (*PDK4*), which inhibits chondrocyte proliferation and migration and accelerates apoptosis. *In vivo* inhibition of *miR-106b* improves arthritic symptoms in a CIA model (Liu et al., 2020). On the other hand, the researcher also found that *in vitro* osteoblasts can phagocytose *miR-486-5p* of exosomes originating from RA FLS and increase miRNA expression, which inhibits transducer of erb-b2 receptor tyrosine kinase 2, 1(*TOB1*) and activates the bone morphogenetic protein (BMP)/suppressor of mothers against decapentaplegic (SMAD) pathway to promote osteoblast differentiation, which is potentially beneficial in RA treatment (Chen et al., 2020).

## Potential effects of exosomes from different sources on macrophages in RA

Research on exosomes from different sources for their effects on macrophages in RA is still relatively limited. Secretion of *miR-let-7b*-containing exosomes by macrophages themselves promotes the differentiation of M1-type pro-inflammatory macrophages in RA joint inflammation by binding Toll-like receptor 7 through the GU structural domain (Kim et al., 2016). Additionally, serum-derived exosome *miR-6089* and *miR-548a-3p* can regulate macrophage proliferation and differentiation, as well as inflammatory factor production, by targeting Toll-like receptor 4 (TLR4) (Wang et al., 2018; Xu et al., 2019). This is also in line with the pro-inflammatory role of macrophages themselves. miR-223 from exosomes sourced from bone marrow mesenchymal stem cells inhibits the release of IL-1β, TNF-α, IL-18 and the activation of the NOD-like receptor protein 3 (NLRP3) to attenuate macrophage inflammation (Huang et al., 2022c). Notably, a new type of nanovesicle was made by combining the membrane of an M1 macrophage with nanovesicles that mimic exosomes from M2 macrophages (Zhao et al., 2022). The nanovesicle has the ability to effectively reduce inflammation by binding to proinflammatory molecules and releasing anti-inflammatory mediators (Zhao et al., 2022). This comprehensive anti-inflammatory activity makes it a powerful tool for treating inflammatory diseases such as rheumatoid arthritis (Zhao et al., 2022) Figure 1.

**FIGURE 1:**
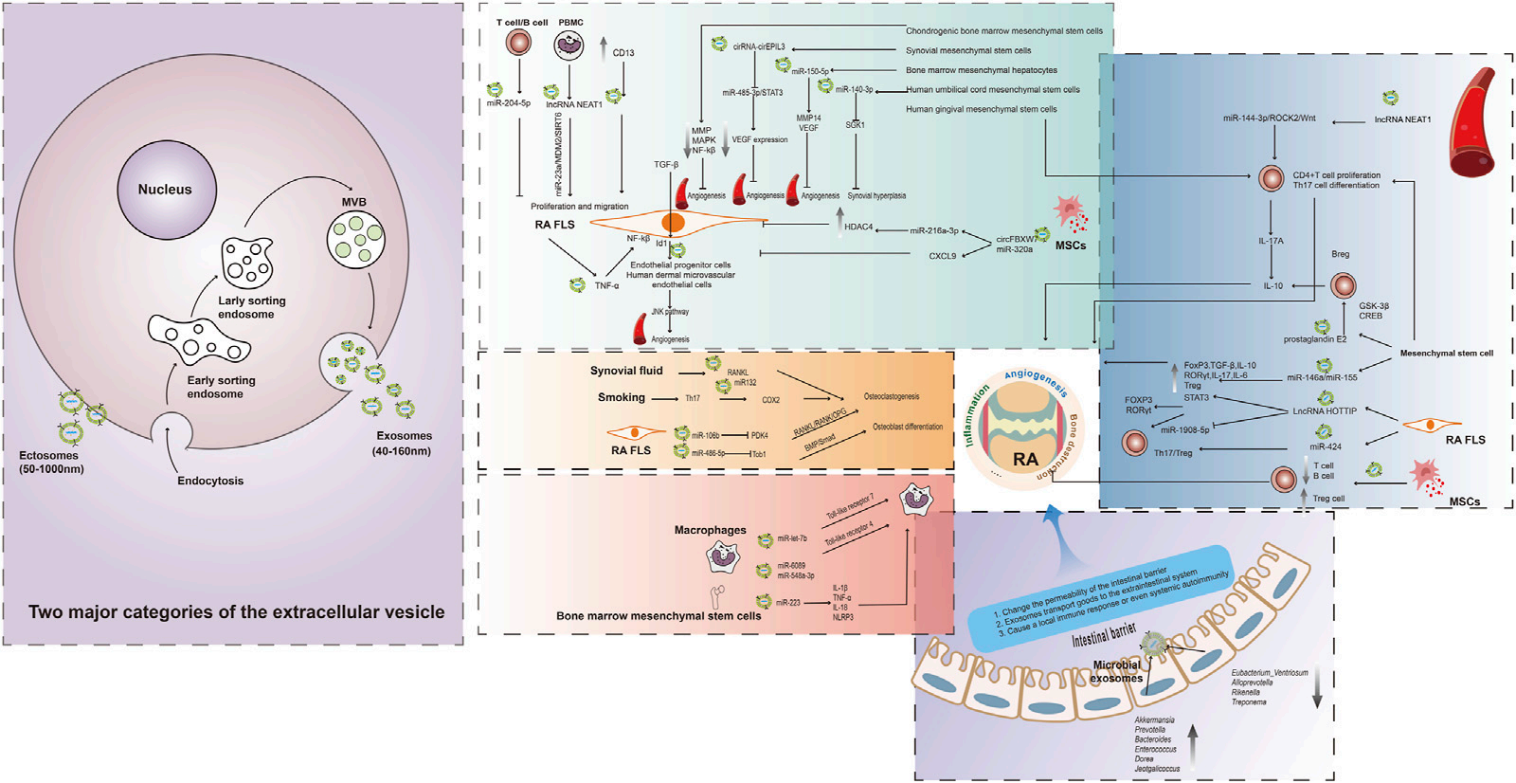
Association of multiple exosomes with RA. The extracellular vesicle can primarily be classified into two major categories, namely, ectosomes and exosomes. Ectosomes represent vesicles that result from the direct outward protrusion of the plasma membrane, generating entities such as microvesicles, microparticles, and large vesicles with sizes. The production of exosomes involves a double invagination of the plasma membrane and the creation of intracellular multivesicular bodies (MVBs) containing intraluminal vesicles (ILVs). The ILVs are eventually released as exosomes through the process of MVB fusion with the plasma membrane and subsequent exocytosis. Blood, FLS, mesenchymal stem cells, and other cell-derived exosomes are all associated with RA through different mechanisms. Gut microbe-derived exosomes are mainly involved in altering the permeability of the intestinal barrier and transporting cargo to the extraintestinal system affecting the immune system, and may be a novel target for future RA therapy.

## The association of gut microbes with RA

The intestine is the organ with the largest surface area in the body. Various microorganisms are colonized in the intestine, and these microbial communities and their products play a vital role in the immune system (Round and Mazmanian, 2009). The pathophysiological relevance of gut microbes in RA may vary (Table 1). The possible mechanism by which gut microbes influence autoimmunity is as follows:

(i). Intestinal barrier dysfunction: Intestinal barrier dysfunction caused by intestinal microbial disorders is an important checkpoint for inflammation in CIA models and RA patients, and the use of the intestinal barrier modulator butyrate can effectively inhibit arthritis (Tajik et al., 2020). There are abnormal changes at the phylum or genus level in various microorganisms in RA. For example, the α- and β-diversity indices of the microbiome are significantly reduced in mice with experimental RA, and they have an elevated proportion of *Firmicutes* and *Bacteroidetes.* Four core genera, including *Eubacterium_Ventriosum, Alloprevotella, Rikenella,* and *Treponema,* were reduced in fecal and mucus content (Nguyen et al., 2022).
(ii). Molecular mimicry, in which gut microbes or metabolites trigger an autoimmune response of immune cells. The epitopes of *Prevotella sp*., *Parabacteroides sp*., and *Butyricimonas sp*. have obvious homology sequence with two autoantigens, N-acetylglucosamine-6-sulfatase, and filamin A, presented by major histocompatibility complex II cell surface receptor in RA (Pianta et al., 2017).Pattern recognition receptors on the surface of antigen-presenting cells constantly recognize and respond to potential antigens in the intestinal lumen, which often include some autoantigens. For example, antigen-presenting cells recognize gut microbes and metabolites (such as LPS and polysaccharide analogs) as potential antigens, leading to downstream T cell cascade differentiation and immune responses, which may initially result in local immunity. However, they can cause systemic autoimmune responses with lymph nodes or humoral circulation (Horta-Baas et al., 2017).
(iii). Regulation of multiple immune cells by multiple metabolites derived from intestinal microorganisms: mainly short-chain fatty acids (butyrate, acetate) and amino acid metabolites (tryptophan metabolism), which can also affect systemic immunity through abnormal intestinal barrier function into the circulation, and the detailed mechanisms can be found in the excellent review (Yang and Cong, 2021). In addition to changes in the microbial community, there were changes in its primary metabolites (short-chain fatty acids, bile acids, amino acids, and tryptophan metabolites) in RA. For example, the *Firmicutes/Bacteroidetes* ratio was low at the gate level in experimental arthritic mice with adjuvant induced arthritis (AIA). At the genus level, there was an abundance of *Akkermansia, Prevotella, Bacteroides, Enterococcus, Dorea, and Jeotgalicoccus* and a lower abundance of *Oscillospira.* Metabolites included acetic acid, propionic acid, butyric acid, valeric acid, capric acid, lactic acid, primary bile acids, secondary bile acids, conjugated bile acids, lipopolysaccharides (LPS), and conjugated bile acids (Cheng et al., 2022).

**TABLE 1:** Abnormally altered microbial communities in RA.

Models	Increase	Potential role of microbial increase	Decrease	Potential role of microbial reduction	Ref
Mice susceptible to collagen-induced arthritis (CIA)	The abundance of OTUs affiliated with the families *Bacteroidaceae*, *Lachnospiraceae*, and *S24-7* increased significantly in CIA-susceptible mice. The abundance of *Desulfovibrio*, *Prevotella, Parabacteroides, Odoribacter, Acetatifactor, Blautia, Coprococcus,* and *Ruminococcus* were enhanced in arthritic mice		*Desulfovibrionaceae* and *Lachnospiraceae* were less abundant in CIA-susceptible mice. *Enterorhabdus, Myroides, Rikenella, Brochothrix, Lactococcus,* and *Streptococcus* were less abundant in arthritic mice		Liu et al. (2016b)
IL-1 receptor antagonist-knockout (IL1rn−/−) mice, IL1rn−/−Tlr2^−/−^ mice, IL1rn−/−Tlr4^−/−^ mice	*Lactobacillus bifidus*	Colonization can lead to rapid development and flare-ups of arthritis			Abdollahi-Roodsaz et al. (2008)
arthritis-susceptible transgenic mice/genetically resistant transgenic mice	*Clostridia* (genus level)	Increased intestinal permeability and a Th17 profile in susceptible mice	*Porphyromonadaceae* and *Bifidobacteriaceae* (the family level)		de Oliveira et al. (2017)
Patients with RA	*Prevotella copri*	*P. copri* dominated the intestinal microbiota and resulted in an increased sensitivity to inflammation			Scher et al. (2013)
RA and healthy controls	*Lactobacillus salivarius, Lactobacillus iners* and *Lactobacillus ruminis*	Potential association with the progression of inflammation			Liu et al. (2013)
RA and healthy controls	*Eggerthella, Actinomyces, Turibacter, Streptococcus,* and *Collinsela* (at the genus level)	Positive correlations with the pro-inflammatory cytokine IL-17			Chen et al. (2016)
RA and non-arthritic controls	*Clostridiaceae*(the family level)	Its increase was found to be characteristic of the arthritic phenotype			Muñiz Pedrogo et al. (2019)
Patients with RA and patients with osteoarthritis	*Bacilli* and *Negativicutes*(the class level), *Selenomonadales* and *Methanobacteriales*(the order level)*, Selenomonadaceae*(the family level), *Megamonas* and *Catenibacterium*(the genus level), *Megamonas funiformis, Clostridium xylanolyticum, Prevotellamassilia timonensis, Catenibacterium mitsuokai, Acidaminococcus fermentans,* and *Bacteroides clarus*(the species level)	Potential stimulation of Th17 and inhibition of Treg cell activity to promote inflammation	*Bacteroides, Bifidobacterium, Bacteroidetes/Firmicutes, Fusicatenibacter saccharivorans, Dialister invisus, Clostridium leptum, Bacteroides faecichinichillae, Bacteroides acidifaciens* and *Christensenella minuta* (at the species level)	Potential Th17 stimulation and Treg cell activity inhibition to promote inflammation	Lee et al. (2019)
Patients with RA and healthy controls	*Bacteroides, Escherichia-Shigella, Parasutterella, Flavonifractor, the Eubacterium xylanophium group, Tyzzerella, Sellimonas, Oscillospira* (at the genus level)	The pathway related to steroid hormone biosynthesis, which includes sex hormones, was enriched in *Bacteroides*	*Lactobacillus, Alloprevotella, Enterobacter, Clostridium sensu stricto-3, Ruminococcaceae UCG-014, Odoribacter, Rikenellaceae RC9, Enterococcus, Klebsiella, Desulfovibrio, Citrobacter, Akkermansia, Helicobacter, Rikenella, Staphylococcus, Coprococcus 1, Coriobacteriaceae UCG-002, Rhodococcus* (at the genus level)	*Alloprevotella* and *Parabacteroides* were positively correlated with the erythrocyte sedimentation rate, and *Prevotella-2* and *Alloprevotella* were positively correlated with C-reactive protein, both biomarkers of inflammation	Sun et al. (2019)
Patients with RA and healthy controls	*Prevotella (P. denticola, P. marshii, P. disiens, P. corporis and P. amnii)*	Th17 cell activation			Kishikawa et al. (2020)
Patients with RA and healthy controls	*phylum Verrucomicrobiae* and genus *Akkermansia*	Association with inflammatory parameters as well as ACPA seropositivity			Chiang et al. (2019)
Pre-clinical RA and controls	*Prevotellaceae*, particularly *Prevotella spp*	Th1 cellular immune response against *P. copri*			Alpizar-Rodriguez et al. (2019)
Treatment-naive individuals with RA and unrelated healthy controls; treatment-naive individuals with RA paired with healthy relatives; and DMARD-treated individuals with RA	*Lactobacillus salivarius*	Positive correlation with titers of the major serum immunoglobulin and IgG	*Haemophilus spp*	Negative correlation with titers of the RA-specific autoantibodies anti-cyclic citrullinated peptide (anti-CCP) and rheumatoid factor (RF)	Zhang et al. (2015)
Patients with RA and healthy controls	*Bacteroides* and *Prevotella species*	Positive correlation with RF	*Clostridium leptum*	Positive correlation with C-reactive protein levels and DAS28-CRP-3 score	Rodrigues et al. (2019)

Abnormal responses to RA can be ameliorated by interventions to improve abnormal microbial changes including some RA clinical agents. For example, increased beneficial microbiota in patients with RA treated with the anti-tumor necrosis factor antibody etanercept (Picchianti-Diamanti et al., 2018). Methotrexate (MTX) may predominantly affect *Bacteroidetes*, and transplantation of microorganisms from patients with RA receiving MTX into germ-free mice under inflammatory stimuli results in a reduced inflammatory response, including reduced activation of multiple immune cells (Nayak et al., 2021). Therefore, correcting the abnormal changes in gut microbiota and metabolites in RA is one of the important research directions of the gut-joint axis. It should be noted that exosomes derived from gut microbiota may be an important component of the gut-joint axis, but as relevant research is still relatively scarce, we will speculate and prospect on the relevant theoretical basis in the following, in anticipation of providing a theoretical basis for future experimental research.

## Future perspectives: gut microbe-derived exosomes may serve as a critical link in the gut-joint axis

Gut microbe-derived exosomes have been suggested as vital mediators of immune transmission in the intestinal mucosa (Kaparakis-Liaskos and Ferrero, 2015; Olsen and Amano, 2015). There are four main types of exosomes derived from intestinal microorganisms: outer membrane vesicles, outer inner membrane vesicles, explosive outer membrane vesicles produced by gram-negative bacteria, and cytoplasmic membrane vesicles produced by gram-positive bacteria (Zhang et al., 2022). In regards to how exosomes from gut microbiota participate in the gut-joint axis, we have the following speculations:

(i). The intact intestinal barrier can prevent excessive activation of the mucosal immune system and inflammatory responses in the intestine (1). For example, exosomes secreted by *the probiotic E. coli strain Nissle 1917* or *commensal E. coli strain ECOR12* can promote the secretion of various inflammatory cytokines and chemokines, including IL-6, IL-8, TNF-α, and chemokine (C-C motif) ligand 3 (CCL3), from peripheral blood mononuclear cells *in vitro* and were unable to elevate these factors in an *in vitro* model of intestinal mucosa co-cultured with Caco-2/peripheral blood mononuclear cells, suggesting that this intact intestinal barrier prevents transitory activation of intestinal mucosal immunity and inflammatory responses (Fábrega et al., 2016).

The gut microbiota and its metabolic products can exert their effects beyond the intestinal area through various complex mechanisms. For example, studies have reported that probiotic intake can improve abnormal disease behaviors through communication from the peripheral immune system to the brain, such as immobility, which is commonly seen in patients with RA, by mechanisms that may involve reducing the number of TNF-α signals in the peripheral immune system that are recruited to monocytes in the brain in response to inflammation and the subsequent activation of brain microglia (D'Mello et al., 2015). Complicated lung disease is a leading cause of death in patients with RA. s*egmented filamentous bacteria* (*SFB*) induce pulmonary autoimmunity by amplifying Th17 cells expressing dual T cell receptors, recognizing their antigenic epitopes, and inducing pulmonary injury in combination with pulmonary expression of C-C motif chemokine ligand 17 (CCL17) (Bradley et al., 2017).

Disturbances in the microbial communities or metabolic can lead to local inflammatory responses in the intestine and increase intestinal permeability (Alghamdi et al., 2021), which may contribute to the systemic diffusion of autoantibodies and the inflammatory response of the joints in RA. For example, the overall gut microbial diversity was reduced in patients with RA and correlated with disease duration and autoantibody levels, and *Collinsella* was significantly associated with the production of pro-inflammatory cytokines and altered intestinal permeability (Chen et al., 2016). In addition, the gut microbial metabolite short-chain fatty acid butyrate was reduced in patients with RA and animal models of arthritis, which may also lead to altered intestinal permeability (Rosser et al., 2020).

(ii). Autoantibody production is a distinctive characteristic of RA, and these autoantibodies may form immune complexes in the joints, leading to chemokine and cytokine secretion through complement activation or direct activation of immune cells, thereby attracting immune cells and enhancing the immune response, ultimately resulting in chronic inflammation and bone destruction (van Delft and Huizinga, 2020).The intestine may be an essential source of autoantibody production. Gut microbe-derived exosomes can be found in tissues and blood throughout the body and interact with multiple immune cells, suggesting that gut-derived exosomes can act as communicators between the intestinal and parenteral systems (Jones et al., 2020; Bittel et al., 2021). They may act as carriers to transport a variety of substances to extraintestinal systems, including joints. When multiple cells release intracellular mitochondrial DNA due to injury, dendritic cells can recognize this DNA to drive Th1 cell polarization in autoimmunity. Exosomes derived from intestinal microbes may initiate an immune response by transporting bacterial DNA, leading to the recognition of CpG motifs by dendritic cells (Lin and Zhang, 2017). In addition, there is a significant increase in gram-negative bacteria in RA, which induces the activation of Th17 cells and immune responses to autoantigens. This is also consistent with the hypothesis of an additional RA-gut link, in which toxic metabolites secreted by intestinal gram-negative bacteria enter the circulation, leading to systemic immunity and autoinflammation (Brusca et al., 2014; de Oliveira et al., 2017). Therefore, exosomes derived from intestinal microbes may also serve as important carriers for transporting self-antibodies produced in the gut to other parts of the body, leading to the inflammatory and bone-destructive processes. Research suggests that synovial-derived exosomes in RA can transport self-antibodies to enhance the autoreactive immune response through the process of antigen presentation (Alghamdi et al., 2021). The symptoms of arthritis in mice were greatly reduced when exposed to sterile conditions, in conjunction with a notable decrease in levels of serum autoantibody titers, splenic autoantibody-secreting cells, and Th17 cells. However, the loss of Th17 cells and autoantibody production in the lamina propria of the small intestine was restored when a single *SFB* was introduced (Wu et al., 2010).

In addition, miRNA-microbe interactions have been explored as a crucial mechanism in various diseases, and miRNA expression patterns in mouse and human fecal and intestinal lumen contents have also been explored (Liu et al., 2016a). Among them, gut microbe-derived exosomes can act as communication mediators to regulate host gene expression and even affect the microbial community (Liu et al., 2016a; Dong et al., 2019; Tavasolian and Inman, 2021). For example, transplantation of gut microbe-derived exosomal miRNAs from wild-type mice ameliorates intestinal barrier dysfunction and inflammation in systemic lupus erythematosus (Liu et al., 2016a). In the context of RA, miRNAs occupy an important position (Chang et al., 2022) and may also interact with the gut-joint axis through exosomes derived from gut microbes.

(iii). The link between gut microbes and joint inflammation has been established and in conjunction with the role of exosomes in RA, we speculate that gut microbe-derived exosomes are involved in the gut-joint axis:

As previously mentioned, there are pathological changes in gut microbiota in RA, including alterations in microbiota abundance and composition, which may result in the production of abnormal bioactive substances (such as metabolites), and impact the pathological features of RA in areas beyond the gut through various mechanisms (such as impaired intestinal barrier function). The abnormal changes in gut microbiota may transmit these aberrant bioactive substances through extracellular vesicles and lead to the subsequent progression of inflammation, angiogenesis, and bone destruction in RA, but further experiments are needed to investigate this phenomenon.

## Discussion

Different sources of exosomes may affect various immune cells in RA through multiple mechanisms, primarily including FLS, T cells, macrophages, osteoclasts, osteoblasts, and chondrocytes. The effects of exosomes from different sources on immune cells depend on the types and functions of the cargoes they carry. It is noteworthy that exosomes derived from mesenchymal stem cells appear to have an overall beneficial effect on RA, which is a promising direction for future development. Abnormal changes in the intestinal flora and multiple sources of exosomes have been observed to have multiple effects on the pathophysiological processes of RA. Although the quantitative proportions of gut microbiota, the most extensive symbiotic system in the human body, are abnormally altered in RA with specific alterations at the phylum and genus levels, metabolites have also been intensively studied and have given birth to many hypotheses. There are still many issues that need to be further elucidated, for example, to clarify the clear linkage of one or several gut-joint axes, widely demonstrated by experiments; nevertheless, there is no doubt that the primary principle is to maintain a reasonable balance between the microbial community and the organism and to prevent ecological imbalances, which may prevent harmful consequences in the gut as well as in the extraintestinal system. Based on the present hypothesis, using samples of preclinical models and clinical patients combined with proteomics, metabolomics, metagenomics technologies, and bioinformatics are essential tools for analysis. Finally, adopting multiple basic molecular biologies and microbiology approaches for continued validation is a necessary direction for the future. Due to the current technical limitations of exosomes, there are still various limitations for their isolation, extraction, and classification, which are proposed to be solved in the future. This is a blank field in the field of RA for microbe-derived exosomes. Gut-derived exosomes may serve as a crucial bridge between gut microbiota and RA. Nevertheless, we have provided reasonable hypotheses on the link between microbe-derived exosomes and the disease based on the association of microbial communities and exosomes with RA. Unfortunately, current relevant studies do not provide sufficient depth; therefore, we can only make appropriate hypotheses on the mechanism of action based on existing studies, mainly related to the alteration of intestinal permeability and possible transport of cargo to extraintestinal systems. Further experimental corroboration is required for in-depth studies. The future direction of our team is to build on this knowledge and gradually enhance future strategic research on the gut-joint axis, which is of great economic and clinical value.

## Figures and Tables

**Table:** 

**RA**	rheumatoid arthritis
**anti-CCP**	anti-cyclic citrullinated peptides
**MISEV**	Minimal Information for Studies of Extracellular Vesicles
**mRNA**	messenger RNA
**ncRNA**	non-coding RNA
**FLS**	fibroblast-like synovial cells
**TNF-α**	tumor necrosis factor-α
**NF-KB**	nuclear factor kappa-light-chain-enhancer of activated B cells
**MMP1**	membrane-type matrix metalloproteinase 1
**CD**	cluster of differentiation
**TGF-β**	transforming growth factor beta
**JNKs**	c-Jun N-terminal kinases
**NEAT1**	nuclear-enriched abundant transcript 1
**MDM2**	mouse double minute 2 homolog
**SIRT6**	sirtuin 6
**VEGF**	vascular endothelial growth factor
**STAT3**	signal transducer and activator of transcription 3
**MSCs**	mesenchymal stem cells
**SGK1**	silencing serum- and glucocorticoid-induced kinase 1
**HDAC4**	histone deacetylase 4
**CXCL9**	C-X-C motif chemokine 9
**MAPK**	mitogen-activated protein kinase
**MMP14**	membrane-type matrix metalloproteinase 14
**CIA**	collagen-induced arthritis
**Th17**	T helper 17
**Treg**	the regulatory T
**IL-17**	Interleukin-17
**HOTTIP**	HOXA distal transcript antisense RNA
**FOXP3**	forkhead Box P3
**RORγt**	retinoid-related orphan receptor gamma-t
**Breg**	regulatory B
**GSK-3 beta**	glycogen synthase kinase-3 beta
**RANKL**	receptor activator of nuclear factor kappa-B ligand
**COX2**	cyclooxygenase-2
**OPG**	osteoprotegerin
**PDK4**	pyruvate dehydrogenase kinase, isozyme 4
**TOB1**	transducer of erb-b2 receptor tyrosine kinase 2, 1
**BMP**	bone morphogenetic protein
**SMAD**	suppressor of mothers against decapentaplegic
**TLR4**	Toll-like receptor 4
**NLRP3**	the NOD-like receptor protein 3
**AIA**	rat adjuvant arthritis
**LPS**	lipopolysaccharides
**MTX**	methotrexate
**SFB**	segmented filamentous bacteria
**CCL17**	C-C motif chemokine ligand 17
**CCL3**	chemokine (C-C motif) ligand 3
**ROCK2**	Rho-associated coiled-coil containing protein kinase 2
